# Dancing Styles of Collective Cell Migration: Image-Based Computational Analysis of JRAB/MICAL-L2

**DOI:** 10.3389/fcell.2018.00004

**Published:** 2018-02-05

**Authors:** Ayuko Sakane, Shin Yoshizawa, Hideo Yokota, Takuya Sasaki

**Affiliations:** ^1^Department of Biochemistry, Tokushima University Graduate School of Medical Sciences, Tokushima, Japan; ^2^Image Processing Research Team, RIKEN Center for Advanced Photonicsm RIKEN, Wako, Japan

**Keywords:** rab GTPases, JRAB/MICAL-L2, conformational change, collective cell migration, computational analysis, optical flow

## Abstract

Collective cell migration is observed during morphogenesis, angiogenesis, and wound healing, and this type of cell migration also contributes to efficient metastasis in some kinds of cancers. Because collectively migrating cells are much better organized than a random assemblage of individual cells, there seems to be a kind of order in migrating clusters. Extensive research has identified a large number of molecules involved in collective cell migration, and these factors have been analyzed using dramatic advances in imaging technology. To date, however, it remains unclear how myriad cells are integrated as a single unit. Recently, we observed unbalanced collective cell migrations that can be likened to either precision dancing or *awa-odori*, Japanese traditional dancing similar to the style at Rio Carnival, caused by the impairment of the conformational change of JRAB/MICAL-L2. This review begins with a brief history of image-based computational analyses on cell migration, explains why quantitative analysis of the stylization of collective cell behavior is difficult, and finally introduces our recent work on JRAB/MICAL-L2 as a successful example of the multidisciplinary approach combining cell biology, live imaging, and computational biology. In combination, these methods have enabled quantitative evaluations of the “dancing style” of collective cell migration.

## Collective cell migration

Cell migration is a fundamental cellular function involved in various biological processes. Broadly speaking, there are two kinds of cell movement: single-cell and collective migration. In the latter, cells form a cluster via adhesion and move as a group. The cells at the migrating front generate a strong force that pulls the mass behind them, whereas the rear cells maintain adhesion and communicate with their neighbors. At the tip of the multicellular group, complete-epithelial to mesenchymal transition (EMT) or partial-EMT is often observed, resulting in the migration mode switch between single-cell and collective cell migration (Friedl and Wolf, 2010; Friedl and Alexander, 2011). In another mode of cell migration with intermediate phenotype, individual cells move one after each other using the same path, which is referred to as multicellular streaming (Friedl and Wolf, 2010; Friedl and Alexander, 2011). Collective cell migration is observed during morphogenesis, angiogenesis, and wound healing (Friedl and Gilmour, 2009; Gray et al., 2010; Mayor and Carmona-Fontaine, 2010; Rørth, 2012; Theveneau and Mayor, 2012) and contributes to efficient metastasis in some kinds of cancers (Sahai, 2005; Friedl and Gilmour, 2009). In other words, this type of cell migration is important in both physiological and pathological contexts. Thus, it has been extensively studied in the field of cell biology (Ladoux and Mège, 2017) and simulation-based biophysics (Ionides, 2001; Woods et al., 2014; Te Boekhorst et al., 2016; George et al., 2017; van Helvert et al., 2018). Collectively migrating cells are much better organized than a random assemblage of individual cells, indicating that one or more factors imposes an order that allows myriad cells to behave as a single unit. Collective cell migration involves multiple molecules [including the Rho family of small GTPases, which regulates actin cytoskeletal reorganization; the Rab family of small GTPases, which regulates membrane trafficking; and extracellular signal-related kinase (ERK), a component of the ERK/MAPK signaling pathway], which are predicted to engage in a complex interplay; Rac and Rho, two members of the Rho family, along with the regulators of their activity, GDP/GTP exchange proteins and GTPase-activating proteins, spatiotemporally regulate actomyosin contractility in moving cell groups (Zegers and Friedl, 2014). Rab5 and Rab11, two members of the Rab family, control the level of Rac activity via intracellular trafficking of receptor tyrosine kinases, resulting in the organization of individual cells during collective cell migration (Ramel et al., 2013). Intracellular propagation waves of ERK activation are observed in groups of cells during collective cell migration (Aoki et al., 2017). The waves induce collective cell migration in the opposite direction. In addition, advances in imaging technology have helped to understand the regulatory systems underlying the complex, higher-order cell functions involved in collective migration. Even with these, however, the whole picture of the regulatory system has not been fully elucidated.

## Image-based computational analysis on cell migration

Various computational tools have been employed for quantitative studies of cell migration. Due to rapid advances in live-cell imaging technology, such data-driven approaches have become popular and important tools for understanding migration dynamics. Model-based analyses, such as statistical or mechanical simulations for directional cell migration (Ionides, 2001; Woods et al., 2014; George et al., 2017), also rely on analysis of data, including images, for verification and calibration of models and parameters.

Although the purpose of quantitative analysis strongly depends on the individual research subjects, useful intermediate information computed from cell migration images can be abstracted for various biological purposes. Hence, image-based analysis yields statistical information about the geometry, topology, and kinematics of individual cells or cell groups, including spatial and temporal descriptors (Cordelières et al., 2013) such as shape, velocity, and motion pattern. Computational frameworks designed to obtain such information usually involve registration/calibration of imaging, filtering (noise reduction, restoration, super-resolution, etc.), segmentation and tracking of cells (or target fluorescent tags), and motion estimation/analysis. For image-based motion analyses of collective cell migration dynamics, the difficulties lie in the segmentation and tracking approaches. The reader is directed to excellent surveys (Castañeda et al., 2014; Masuzzo et al., 2016) for further information about general computational methods and software packages applied to analyses of cell migration.

Motion analysis based on segmentation and tracking approaches has been often applied to phase-contrast and fluorescence images of cell migration, which visualize the cytoplasm, nuclei, or plasma/nuclear membranes. For example, trajectories of individual cell nuclei have been studied in *Drosophila* gastrulation (Supatto et al., 2009), embryogenesis in zebrafish (Khairy and Keller, 2011), and the *Drosophila* border cell system (Cliffe et al., 2017) by linking segmented nuclear regions in adjacent time-lapse images. At the cellular and subcellular scales, the cell and its nucleus are both topologically equivalent to a sphere (i.e., a topological disk/ball), except during cell division. Therefore, finding a pair of corresponding segmented cells/nuclei in adjacent time-lapse images is easier than in cases with no topological restrictions. The ellipsoidal shapes of cells and nuclei in collective cell migration are also favorable for region extraction. Thus, popular unsupervised segmentation methods, such as discriminant analysis (HUVEC: human umbilical vein endothelial cells Huang et al., 2012), active contours (monolayer of cultured pig epithelial cells Bunyak et al., 2006), mean shift [HUVEC, astrocytoma, melanoma, and colon carcinoma cells (Debeir et al., 2005) and human melanoma cells (Cordelières et al., 2013)], and supervised machine learning techniques (Masuzzo et al., 2016) have been employed for motion analysis. The mathematical and algorithmic aspects of these methods were imported from computer science, especially computer vision, pattern recognition, and image processing, and have been adapted to processing of migration images in cell biology. Unfortunately, objects (organelles, cytoskeleton, structures on plasma/nuclear membranes such as pores and receptors, and proteins of interest) in cell images are usually much more complex, and undergo spatiotemporal changes in both their geometry and topology. Objects of this type have not been extensively examined by conventional computer science. In addition, because manually generating sets of teaching images is tedious and time-consuming, it is difficult to acquire enough teaching images containing segmented/tracked regions for use with state-of-the art deep learning techniques. Also, once a training set has been obtained then automatic segmentation (and tracking) based on machine learning techniques could become irrelevant, as quantitative information could be obtained from the teaching images. Although some *a priori* knowledge about cell migration can be incorporated into these computations, this valuable knowledge is the very information that we hope to obtain from image-based computational analysis in the first place. Thus, segmentation and tracking approaches are limited in terms of their applicability for tags for objects other than the cytoplasm and nucleus, such as intracellular structures (hereafter, referred to as general-target tags), especially in the analysis of collective cell migration.

**Figure 1:**
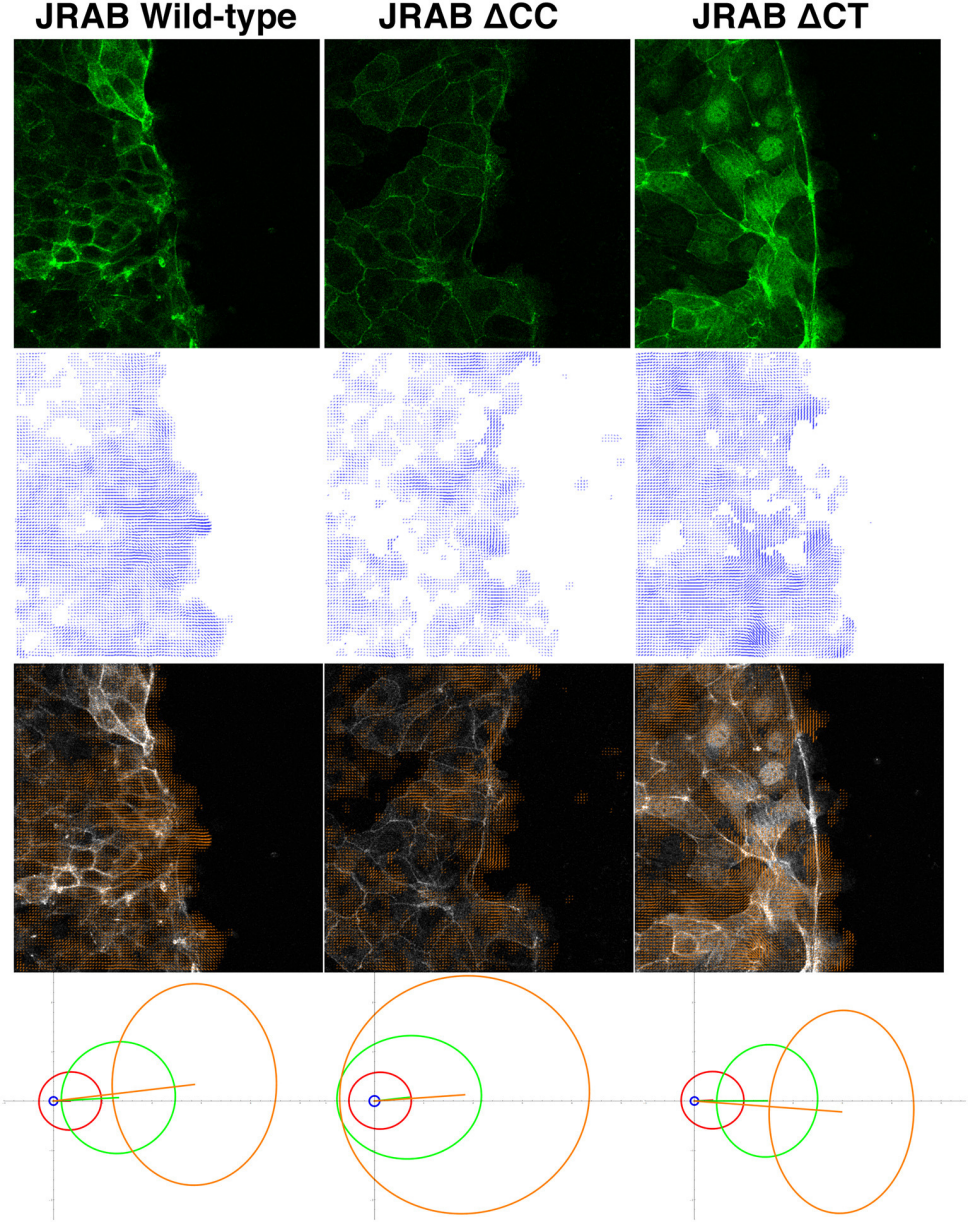
Example of OF analysis of collective cell migration. Top row: live-cell images of cell groups expressing GFP-tagged JRAB wild-type and two mutants. Second row: estimated velocity fields obtained via OF, corresponding to the images in the top row. Third row: overlays of the top and second-row images. Fourth row: PCA results (four different subsets of velocity vectors) corresponding to the datasets represented by the top-row images. More than two billion vectors extracted from 27 time-lapse sets consisting of 7,700 images were employed in the PCA. Each ellipse shows how magnitude and direction of the corresponding velocity vector set varies in 2D space. Coordinate origin represents no movement, and increasing distance from the origin indicates greater velocity. Hence, each ellipse in JRABwt (bottom-left image), especially the two ellipses in the high-speed region, is more concentrated toward the left, which is the direction of cell migration. Thus, JRABwt is more efficient than the other mutants. For more detailed statistical analysis and discussion, see our recent work (Sakane et al., 2016); these figures were adapted from that paper with permission from ASCB.

Motion estimation without segmentation/tracking of target shapes has been applied to cell migration analysis, e.g., a damped harmonic oscillator model often employed in fluid dynamics and a particle image velocimetry software were applied to extract motion fields of cells (cell populations) in (Angelini et al., 2011) and (Jang et al., 2017), respectively. The most common technique employed in such motion analyses [including intracellular logistics at the Golgi apparatus (Ben-Tekaya et al., 2005)] is Optical Flow (OF), which estimates a motion field consisting of a velocity vector at each pixel of a live-cell image (see middle images of Figure 1 as examples of motion fields with their corresponding live images). Although many OF models have been developed [see (Delpiano et al., 2012) for some of these models applied to point signals in fluorescence images], the general idea is based on the hypothesis that the intensity/texture of local regions in time-varying images is approximately constant under motion, at least over short timescales. This hypothesis leads to the so-called OF constraint equation, consisting of the spatial gradient and temporal first-order partial derivative (speed) of the image intensity; see seminal surveys (Beauchemin and Barron, 1995; Fortun et al., 2015) for more information on mathematical formulation, computational methodology, and applications. Once motion fields are obtained, spatial and temporal descriptors (Castañeda et al., 2014) are usually extracted to represent quantitative and salient features of the target tags, as well as visualizing the vector field along with its corresponding cell migration image. The trajectories of the target tags are obtained by averaging velocity vectors within a local or segmented image region.

## Optical flows in collective cell migration analysis

OF is also popular for analysis of collective cell migration images consisting of cytoplasm or nuclei (Breen and Williams, 1994; Siegert et al., 1994; Ronot et al., 2000; Dubin-Thaler et al., 2008; Amat et al., 2013; Boric et al., 2013; Kappe et al., 2016), despite the fact that segmentation and tracking approaches work well on such images.

Breen and Williams developed an OF-based digital imaging vision system and applied it to ventral cellular layers of the migrating *Dictyostelium discoideum* slug (Breen and Williams, 1994). Their system characterized the speed of the layers and migrating tips of the slug based on velocity profiles and the corresponding cross-correlation analysis. Siegert et al. reported an OF-based image processing method and demonstrated its use on phase-contrast images of the *Dictyostelium* developmental cycle (Siegert et al., 1994). Their analysis, based on velocity profiles, the color-coded velocity map, and cell trajectories, showed that *Dictyostelium* development from the aggregation stage onwards, is governed by rotational movement. Ronot et al. proposed an OF-based image analysis approach and used it to study cell movement in phase-contrast images of HeLa monolayers undergoing wound healing (Ronot et al., 2000). Their approach estimated the velocity profiles and affine transformation coefficients of the monolayers, and then quantified the various phases of the wound repair process and wound closure dynamics in multiple cell lines. Dubin-Thaler et al. studied single-cell motility during cell spreading, based on velocity fraction histograms and auto-correlation of velocity maps calculated from OF-based image analysis (Dubin-Thaler et al., 2008). They proposed that regulatory pathways provide some combination of local motility modules that contribute to the overall motility function of the cell. Boric et al. developed an assay for analyzing and quantifying migration of cranial neural crest cells in zebrafish based on OF-based image analysis (Boric et al., 2013). Specifically, they characterized cell migration patterns of normal and ethanol-exposed embryos, based on directional histograms with a polar coordinate system, hue color visualization of velocity vectors, and displacement factors over time.

OF-based analysis has also been tailored to the specific characteristics of 3D time-lapse light-sheet microscopy datasets, especially for images of the nucleus, e.g., in *Drosophila* and zebrafish development (Amat et al., 2013) and *Drosophila* gastrulation (Kappe et al., 2016). On the other hand, OF is rarely applied to general-target fluorescent tags in collective cell migration analyses, even though (as mentioned above) it is suitable for such images. In contrast to these studies, images of a single molecule called JRAB/MICAL-L2 and its mutants labeled with such general-target tags were quantitatively analyzed using an OF technique, revealing its pivotal role in the “dancing styles” of collective cell migration (Sakane et al., 2016). The rest of the review focuses on JRAB/MICAL-L2 and how it affects migration dynamics.

## JRAB/MICAL-L2 may control collective dynamics

JRAB (Junctional Rab13-binding protein)/MICAL-L2 (molecules interacting with CasL-like 2) is an effector protein of Rab13 (Terai et al., 2006), a member of the Rab family of small GTPases, that serves as a molecular switch in the regulation of membrane trafficking (Takai et al., 2001; Zerial and McBride, 2001; Hutagalung and Novick, 2011). Several studies, including our own, have shown that Rab13–JRAB/MICAL-L2 is involved in the transport of cell adhesion molecules and thereby regulates cell–cell adhesion in epithelia (Zahraoui et al., 1994; Marzesco et al., 2002; Morimoto et al., 2005; Terai et al., 2006; Yamamura et al., 2008; Sakane and Sasaki, 2015). Furthermore, JRAB/MICAL-L2 engages in an intramolecular interaction between its N-terminal and C-terminal regions, and binding of Rab13 releases this interaction, resulting in a conformational change from the closed to open form (Sakane et al., 2010). This change is also involved in the spatiotemporal regulation of actin dynamics during epithelial junctional development (Sakane et al., 2012). A structural model of JRAB/MICAL-L2 based on an approach combining bioinformatics and biochemistry (Sakane et al., 2016) has also provided firm evidence for a conformational change induced by Rab13.

These observations raise the possibility that JRAB/MICAL-L2, through its conformational change, coordinates cell–cell adhesion and individual cell migration during collective cell migration.

## Conformational change of JRAB/MICAL-L2 in the cell

To test the above hypothesis, we generated JRAB mutants fixed in a specific conformation: open form, JRABΔCC; closed form, JRABΔCT (Sakane et al., 2010). Further investigations (Sakane et al., 2016) of individual cells expressing GFP-JRABΔCC or GFP-JRABΔCT provided the following observations and biological results.

JRABΔCT enhanced the formation of the thick F-actin bundle along the free edge. In addition, radial actin filaments extended from the F-actin bundle, resulting in the maturation of focal adhesions. By contrast, neither the thick F-actin bundle nor mature focal adhesions were observed at front cells expressing JRABΔCC. These differences may explain the results of the biomechanical analyses showing that JRABΔCT generates a traction force at the free edge of the cell population, whereas JRABΔCC impairs this force (Sakane et al., 2016).

We also examined the follower cells, and found that the cells expressing JRABΔCT were larger in size at the junctional level than those expressing JRABΔCC. Moreover, in cells expressing JRABΔCT, many more stress fibers were observed at the basal level than in cells expressing JRABΔCC. These findings imply that JRABΔCT shifts the actin cytoskeleton from cell–cell adhesion to cell–matrix adhesion, thereby increasing the area of the cell at the junctional level. On the contrary, JRABΔCC maintains an appropriate cell area at the junctional level, probably via the formation of stable cell–cell junctions. The results of hanging-drop culture assays support the idea that JRABΔCC maintains more stable cell–cell adhesion, resulting in larger spheroids than those formed by control cells or those expressing JRABΔCT. Taken together, these observations indicate that at the front, the closed form of JRAB/MICAL-L2 plays a role in the generation of traction force that pulls the population in a certain direction, whereas the open form of JRAB/MICAL-L2 contributes to formation and maintenance of stable cell–cell adhesion between follower cells, enabling them to behave as a single unit.

## Quantification of “dancing styles” via optical flow

In wound healing assays using epithelial MTD-1A cells expressing these mutants, different conformations of JRAB/MICAL-L2 exhibited distinctive “dancing styles” based on the regulation of actin dynamics (Sakane et al., 2016). Groups of cells expressing GFP-JRABΔCT moved strongly in a fixed direction, akin to precision dancing (top-right image of Figure 1). By contrast, remarkable ruffles were observed along the front line of clusters of cells expressing JRABΔCC, which migrated in random directions (top-center image of Figure 1), resembling *awa-odori*, a Japanese form of traditional dancing similar to the style observed at the Rio Carnival. Groups of cells expressing GFP-JRABwt exhibited an intermediate phenotype (top-left image of Figure 1; Sakane et al., 2016).

Because GFP fusions of wild-type JRAB/MICAL-L2, JRABΔCC, and JRABΔCT are general-target fluorescent tags, as discussed above, OF is suitable for characterizing their associated dancing styles from live images and performing quantitative analyses. Hence, we applied an OF technique to study distinctive features of GFP-tagged wild-type JRAB/MICAL-L2 and its conformational mutants during collective cell migration (Sakane et al., 2016). Principal component analysis (PCA) was applied to a subset of velocity vectors classified by speed magnitude based on the extracted OF motion fields (bottom images of Figure 1); in this context, PCA provided a set of linearly uncorrelated directions and corresponding magnitudes of a set of time-lapse motion fields. We found that the high-speed motions of JRABwt were more concentrated in the direction corresponding to cell migration than those of the mutants. Thus, wild-type JRAB/MICAL-L2, which can change its structure between the open and closed forms, allows cells to behave as an effective unit. By contrast, mutants locked in the open or closed form did not exhibit this behavior.

## Concluding remarks

**Figure 2:**
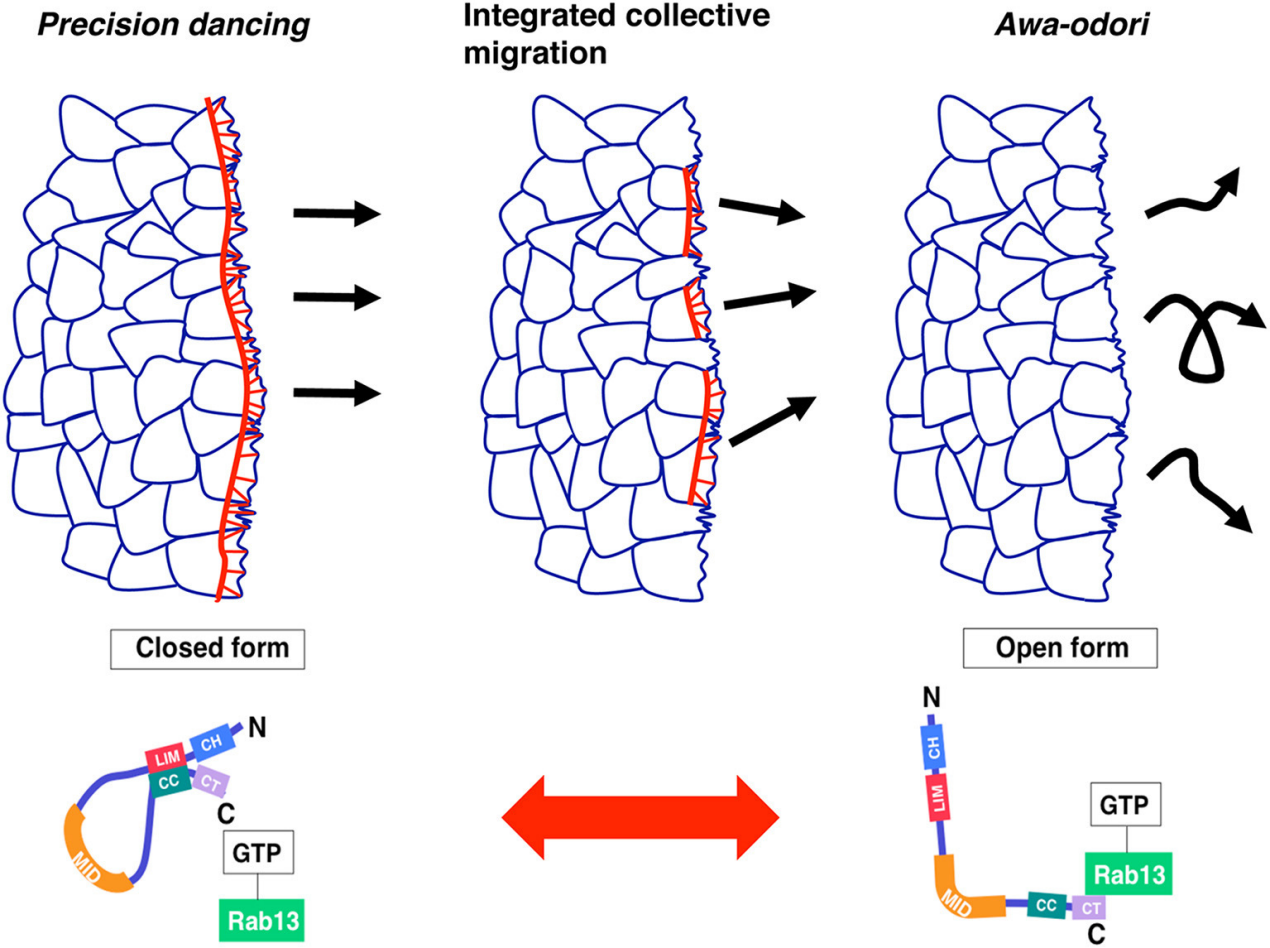
Model: collective cell migration regulated by JRAB/MICAL-L2 conformational plasticity. A cell population expressing only the closed form of JRAB exhibits precision dancing (left), whereas a population expressing only the open form exhibits movement analogous to *awa-odori* (right). For integrated collective cell migration, JRAB/MICAL-L2 must be able to change its conformation freely depending on the situation (middle). This figure was reproduced from our recent work (Sakane et al., 2016) with permission from ASCB.

In this review, two different but linked topics were discussed: image-based computational methods and the functional role of JRAB/MICAL-L2 in collective cell migration. Although JRAB/MICAL-L2 was expected to be a key player in migration dynamics, conventional molecular, and cell biology approaches were not sufficient to establish its importance. To address this issue, we used a robust approach combining cell biology, live imaging, and computational analysis (especially OF methods) to evaluate the “dancing style” of migrating cells expressing JRAB/MICAL-L2 or its variants. The results provided valuable insight into a longstanding question concerning how several cells are organized in a moving cell population. Indeed, recent advances in computational analysis provides us with a simple model in which conformational plasticity of a single molecule, JRAB/MICAL-L2, generates the order underlying the integrated movements associated with collective cell migration (Figure 2).

OF-based methods and software systems will continue to expand in terms of methodology and application (Delpiano et al., 2012; Fortun et al., 2015; Masuzzo et al., 2016). Also integrating the obtained motion fields with model-based computational analyses such as mechanical and biochemical simulations of cell migrations (Te Boekhorst et al., 2016; van Helvert et al., 2018) is useful and promising future work. Consequently, biologists may be able to choose from among existing tools in order to obtain motion fields in their target images. On the other hand, spatial and temporal descriptors in motion field analysis of collective cell migration images remain rather simple in comparison with those developed in computational fluid dynamics, medical image processing, and computer graphics. Therefore, in the future, the more advanced mathematical tools of vector field analysis, such as vector field topology (Theisel et al., 2005; Wang et al., 2013), Hodge decomposition (Bhatia et al., 2013), and ridge creases (Tricoche et al., 2008; Schultz et al., 2010) could be useful, and such sophisticated methods might become popular in cell biology.

## Author contributions

All authors listed have made a substantial, direct and intellectual contribution to the work, and approved it for publication.

### Conflict of interest statement

The authors declare that the research was conducted in the absence of any commercial or financial relationships that could be construed as a potential conflict of interest.

## References

[B1] Amat F. Myers E. W. Keller P. J. (2013). Fast and robust optical flow for time-lapse microscopy using super-voxels. Bioinformatics 29, 373–380. 10.1093/bioinformatics/bts706 23242263 PMC3562071

[B2] Angelini T. E. Hannezo E. Trepat X. Marquez M. Fredberg J. J. Weitz D. A. (2011). Glass-like dynamics of collective cell migration. Proc. Natl. Acad. Sci. U.S.A. 108, 4714–4719. 10.1073/pnas.1010059108 21321233 PMC3064326

[B3] Aoki K. Kondo Y. Naoki H. Hiratsuka T. Itoh R. E. Matsuda M. (2017). Propagating wave of ERK activation orients collective cell migration. Dev Cell 43, 305.e5–317.e5. 10.1016/j.devcel.2017.10.016 29112851

[B4] Beauchemin S. S. Barron J. L. (1995). The computation of optical flow. ACM Comput. Surv. 27, 433–466. 10.1145/212094.212141

[B5] Ben-Tekaya H. Miura K. Pepperkok R. Hauri H. P. (2005). Live imaging of bidirectional traffic from the ERGIC. J. Cell Sci. 118, 357–367. 10.1242/jcs.01615 15632110

[B6] Bhatia H. Norgard G. Pascucci V. Bremer P. T. (2013). The helmholtz-hodge decomposition–a survey. IEEE Trans. Vis. Comput. Graph. 19, 1386–1404. 10.1109/TVCG.2012.316 23744268

[B7] Boric K. Orio P. Vièville T. Whitlock K. (2013). Quantitative analysis of cell migration using optical flow. PLoS ONE 8:e69574. 10.1371/journal.pone.0069574 23936049 PMC3729970

[B8] Breen E. J. Williams K. L. (1994). Optical flow analysis of the ventral cellular layer of the migrating *Dictyostelium discoideum* slug. Microbiology 140 (Pt 5), 1241–1252. 10.1099/13500872-140-5-1241 8025690

[B9] Bunyak F. Palaniappan K. Nath S. K. Baskin T. I. Dong G. (2006). Quantitative cell motility for in vitro wound healing using level set-based active contour tracking, in Proceedings IEEE International Symposium on Biomedical Imaging (Arlington, VA: IEEE), 1040–1043. 10.1109/ISBI.2006.1625099 PMC2705119 19578557

[B10] Castañeda V. Cerda M. Santibáñez F. Jara J. Pulgar E. Palma K.. (2014). Computational methods for analysis of dynamic events in cell migration. Curr. Mol. Med. 14, 291–307. 10.2174/1566524014666140128113952 24467201

[B11] Cliffe A. Doupé D. P. Sung H. Lim I. K. Ong K. H. Cheng L.. (2017). Quantitative 3D analysis of complex single border cell behaviors in coordinated collective cell migration. Nat. Commun. 8:14905. 10.1038/ncomms14905 28374738 PMC5382290

[B12] Cordelières F. P. Petit V. Kumasaka M. Debeir O. Letort V. Gallagher S. J.. (2013). Automated cell tracking and analysis in phase-contrast videos (iTrack4U): development of Java software based on combined mean-shift processes. PLoS ONE 8:e81266. 10.1371/journal.pone.0081266 24312283 PMC3842324

[B13] Debeir O. Van Ham P. Kiss R. Decaestecker C. (2005). Tracking of migrating cells under phase-contrast video microscopy with combined mean-shift processes. IEEE Trans. Med. Imaging 24, 697–711. 10.1109/TMI.2005.846851 15957594

[B14] Delpiano J. Jara J. Scheer J. Ramírez O. A. Ruiz-Del-Solar J. Härtel S. (2012). Performance of optical flow techniques for motion analysis of fluorescent point signals in confocal microscopy. Mach. Vis. Appl. 23, 675–689. 10.1007/s00138-011-0362-8

[B15] Dubin-Thaler B. J. Hofman J. M. Cai Y. Xenias H. Spielman I. Shneidman A. V.. (2008). Quantification of cell edge velocities and traction forces reveals distinct motility modules during cell spreading. PLoS ONE 3:e3735. 10.1371/journal.pone.0003735 19011687 PMC2581916

[B16] Fortun D. Bouthemy P. Kervrann C. (2015). Optical flow modeling and computation: a survey. Comput. Vis. Image Underst. 134, 1–21. 10.1016/j.cviu.2015.02.008

[B17] Friedl P. Alexander S. (2011). Cancer invasion and the microenvironment: plasticity and reciprocity *Cell* 147, 992–1009. 10.1016/j.cell.2011.11.016 22118458

[B18] Friedl P. Gilmour D. (2009). Collective cell migration in morphogenesis, regeneration and cancer. Nat. Rev. Mol. Cell Biol. 10, 445–457. 10.1038/nrm2720 19546857

[B19] Friedl P. Wolf K. (2010). Plasticity of cell migration: a multiscale tuning model. J. Cell Biol. 188, 11–19. 10.1083/jcb.200909003 19951899 PMC2812848

[B20] George M. Bullo F. Campàs O. (2017). Connecting individual to collective cell migration. Sci. Rep. 7:9720. 10.1038/s41598-017-10069-8 28852093 PMC5575354

[B21] Gray R. S. Cheung K. J. Ewald A. J. (2010). Cellular mechanisms regulating epithelial morphogenesis and cancer invasion. Curr. Opin. Cell Biol. 22, 640–650. 10.1016/j.ceb.2010.08.019 20832275 PMC2948645

[B22] Huang G. Kim J. Huang X. Zheng G. Tokuta A. (2012). A statistical framework for estimation of cell migration velocity. J. WSCG 20, 29–36.

[B23] Hutagalung A. H. Novick P. J. (2011). Role of Rab GTPases in membrane traffic and cell physiology. Physiol. Rev. 91, 119–149. 10.1152/physrev.00059.2009 21248164 PMC3710122

[B24] Ionides E. (2001). Statistical Analysis of Cell Motion. Ph.D. thesis, University of Calfornia.

[B25] Jang H. Notbohm J. Gweon B. Cho Y. Park C. Y. Kee S. H.. (2017). Homogenizing cellular tension by hepatocyte growth factor in expanding epithelial monolayer. Sci. Rep. 8:45844. 10.1038/srep45844 28374776 PMC5379206

[B26] Kappe C. P. Schütz L. Gunther S. Hufnagel L. Lemke S. Leitte H. (2016). Reconstruction and visualization of coordinated 3D cell migration based on optical flow. IEEE Trans. Vis. Comput. Graph. 22, 995–1004. 10.1109/TVCG.2015.2467291 26529743

[B27] Khairy K. Keller P. J. (2011). Reconstructing embryonic development. Genesis 49, 488–513. 10.1002/dvg.20698 21140407

[B28] Ladoux B. Mège R. M. (2017). Mechanobiology of collective cell behaviours. Nat. Rev. Mol. Cell Biol 18, 743–757. 10.1038/nrm.2017.98 29115298

[B29] Marzesco A. M. Dunia I. Pandjaitan R. Recouvreur M. Dauzonne D. Benedetti E. L.. (2002). The small GTPase Rab13 regulates assembly of functional tight junctions in epithelial cells. Mol. Biol. Cell 13, 1819–1831. 10.1091/mbc.02-02-0029 12058051 PMC117606

[B30] Masuzzo P. Van Troys M. Ampe C. Martens L. (2016). Taking aim at moving targets in computational cell migration. Trends Cell Biol. 26, 88–110. 10.1016/j.tcb.2015.09.003 26481052

[B31] Mayor R. Carmona-Fontaine C. (2010). Keeping in touch with contact inhibition of locomotion. Trends Cell Biol. 20, 319–328. 10.1016/j.tcb.2010.03.005 20399659 PMC2927909

[B32] Morimoto S. Nishimura N. Terai T. Manabe S. Yamamoto Y. Shinahara W.. (2005). Rab13 mediates the continuous endocytic recycling of occludin to the cell surface. J. Biol. Chem. 280, 2220–2228. 10.1074/jbc.M406906200 15528189

[B33] Ramel D. Wang X. Laflamme C. Montell D. J. Emery G. (2013). Rab11 regulates cell-cell communication during collective cell movements. Nat. Cell Biol. 15, 317–324. 10.1038/ncb2681 23376974 PMC4006229

[B34] Ronot X. Doisy A. Tracqui P. (2000). Quantitative study of dynamic behavior of cell monolayers during *in vitro* wound healing by optical flow analysis. Cytometry 41, 19–30. 10.1002/1097-0320(20000901)41:1<19::AID-CYTO3>3.0.CO;2-X 10942892

[B35] Rørth P. (2012). Fellow travellers: emergent properties of collective cell migration. EMBO Rep. 13, 984–991. 10.1038/embor.2012.149 23059978 PMC3492716

[B36] Sahai E. (2005). Mechanisms of cancer cell invasion. Curr. Opin. Genet. Dev. 15, 87–96. 10.1016/j.gde.2004.12.002 15661538

[B37] Sakane A. Abdallah A. A. Nakano K. Honda K. Ikeda W. Nishikawa Y.. (2012). Rab13 small G protein and junctional Rab13-binding protein (JRAB) orchestrate actin cytoskeletal organization during epithelial junctional development. J. Biol. Chem. 287, 42455–42468. 10.1074/jbc.M112.383653 23100251 PMC3522248

[B38] Sakane A. Sasaki T. (2015). Roles of Rab family small G proteins in formation of the apical junctional complex in epithelial cells, in Cell Polarity ed Ebnet K. (Germany: Springer), 349–374. 10.1007/978-3-319-14463-4_15

[B39] Sakane A. Honda K. Sasaki T. (2010). Rab13 regulates neurite outgrowth in PC12 cells through its effector protein, JRAB/MICAL-L2. Mol. Cell. Biol. 30, 1077–1087. 10.1128/MCB.01067-09 20008558 PMC2815571

[B40] Sakane A. Yoshizawa S. Nishimura M. Tsuchiya Y. Matsushita N. Miyake K.. (2016). Conformational plasticity of JRAB/MICAL-L2 provides “law and order” in collective cell migration. Mol. Biol. Cell 27, 3095–3108. 10.1091/mbc.E16-05-0332 27582384 PMC5063617

[B41] Schultz T. Theisel H. Seidel H. P. (2010). Crease surfaces: from theory to extraction and application to diffusion tensor MRI. IEEE Trans. Vis. Comput. Graph. 16, 109–119. 10.1109/TVCG.2009.44 19910665

[B42] Siegert F. Weijer C. J. Nomura A. Miike H. (1994). A gradient method for the quantitative analysis of cell movement and tissue flow and its application to the analysis of multicellular Dictyostelium development. J. Cell Sci. 107(Pt 1), 97–104. 8175927 10.1242/jcs.107.1.97

[B43] Supatto W. McMahon A. Fraser S. E. Stathopoulos A. (2009). Quantitative imaging of collective cell migration during *Drosophila* gastrulation: multiphoton microscopy and computational analysis. Nat. Protoc. 4, 1397–1412. 10.1038/nprot.2009.130 19745822 PMC2854020

[B44] Takai Y. Sasaki T. Matozaki T. (2001). Small GTP-binding proteins. Physiol. Rev. 81, 153–208. 10.1152/physrev.2001.81.1.153 11152757

[B45] Te Boekhorst V. Preziosi L. Friedl P. (2016). Plasticity of cell migration *in vivo* and *in silico*. Annu. Rev. Cell Dev. Biol. 32, 491–526. 10.1146/annurev-cellbio-111315-125201 27576118

[B46] Terai T. Nishimura N. Kanda I. Yasui N. Sasaki T. (2006). JRAB/MICAL-L2 is a junctional Rab13-binding protein mediating the endocytic recycling of occludin. Mol. Biol. Cell 17, 2465–2475. 10.1091/mbc.E05-09-0826 16525024 PMC1446078

[B47] Theisel H. Weinkauf T. Hege H. C. Seidel H. P. (2005). Topological methods for 2D time-dependent vector fields based on stream lines and path lines. IEEE Trans. Vis. Comput. Graph. 11, 383–394. 10.1109/TVCG.2005.68 16138549

[B48] Theveneau E. Mayor R. (2012). Cadherins in collective cell migration of mesenchymal cells. Curr. Opin. Cell Biol. 24, 677–684. 10.1016/j.ceb.2012.08.002 22944726 PMC4902125

[B49] Tricoche X. Kindlmann G. Westin C. F. (2008). Invariant crease lines for topological and structural analysis of tensor fields. IEEE Trans. Vis. Comput. Graph. 14, 1627–1634. 10.1109/TVCG.2008.148 18989019 PMC2743867

[B50] van Helvert S. Storm C. Friedl P. (2018). Mechanoreciprocity in cell migration. Nat. Cell Biol. 20, 8–20. 10.1038/s41556-017-0012-0 29269951 PMC5943039

[B51] Wang C. Shen H.-W. Weiskopf D. Peterka T. Chen G. (2013). State-of-the-art flow field analysis and visualization. 2013 IEEE VIS Tutorials.

[B52] Woods M. L. Carmona-Fontaine C. Barnes C. P. Couzin I. D. Mayor R. Page K. M. (2014). Directional collective cell migration emerges as a property of cell interactions. PLoS ONE 9:e104969. 10.1371/journal.pone.0104969 25181349 PMC4152153

[B53] Yamamura R. Nishimura N. Nakatsuji H. Arase S. Sasaki T. (2008). The interaction of JRAB/MICAL-L2 with Rab8 and Rab13 coordinates the assembly of tight junctions and adherens junctions. Mol. Biol. Cell 19, 971–983. 10.1091/mbc.E07-06-0551 18094055 PMC2262956

[B54] Zahraoui A. Joberty G. Arpin M. Fontaine J. J. Hellio R. Tavitian A.. (1994). A small rab GTPase is distributed in cytoplasmic vesicles in non polarized cells but colocalizes with the tight junction marker ZO-1 in polarized epithelial cells. J. Cell Biol. 124, 101–115. 10.1083/jcb.124.1.101 8294494 PMC2119893

[B55] Zegers M. M. Friedl P. (2014). Rho GTPases in collective cell migration. Small GTPases 5:e28997. 10.4161/sgtp.28997 25054920 PMC4114924

[B56] Zerial M. McBride H. (2001). Rab proteins as membrane organizers. Nat. Rev. Mol. Cell Biol. 2, 107–117. 10.1038/35052055 11252952

